# Visceral Leishmaniasis or Systemic Lupus Erythematosus Flare?

**DOI:** 10.1155/2012/523589

**Published:** 2012-08-22

**Authors:** Sunny Garg, Mousumi Kundu, Amit Nandan Dhar Dwivedi, Lalit Prashant Meena, Neeraj Varyani, Asif Iqbal, Kamlakar Tripathi

**Affiliations:** ^1^Department of General Medicine, Institute of Medical Sciences, Banaras Hindu University, Varanasi 221005, India; ^2^Department of Radiology, Institute of Medical Sciences, Banaras Hindu University, Varanasi 221005, India

## Abstract

Systemic lupus erythematosus (SLE) is a multisystem disorder characterised by B-cell hyperactivity with production of multiple autoantibodies. Fever in SLE may be caused by disease exacerbation or by infection. We report a patient of SLE that was later complicated by fever, pancytopenia, and massive splenomegaly. Corticosteroid therapy for SLE might have masked the underlying infection at earlier stage. Despite negative results of rk-39 test and bone marrow biopsy, a very high suspicion for visceral leishmaniasis (VL) led us to go for direct agglutination test (DAT) and polymerase chain reaction (PCR) for leishmanial antigen that revealed positive results. Moreover, significant improvement in clinical and biochemical parameters was noted on starting the patient on antileishmanial therapy.

## 1. Introduction

Systemic lupus erythematosus (SLE) is a chronic autoimmune disease of unknown aetiology, characterised by B cell hyperactivity and production of multiple autoantibodies. Haematological abnormalities of SLE include haemolytic anemia, leucopenia or lymphopenia, and thrombocytopenia, due to the production of autoantibodies [1]. Splenomegaly is not a common sign of SLE, unless there is concurrent infection.

On the other hand, visceral leishmaniasis is a chronic parasitic infection caused by *Leishmania donovani*. There is B cell hyperactivity, resulting in production of autoantibodies such as ANA and others [2]. It is characterised by fever, cytopenias, and splenomegaly. Splenomegaly and hypersplenism are mainly responsible for cytopenias. Immunocompromised patients due to acquired immunodeficiency syndrome, after kidney-transplantation or leukemia are more commonly affected [3].

We report a case of SLE complicated by visceral leishmaniasis, with sharp clinical resemblance to a flare of SLE. A high suspicion for kala azar should always be kept when a patient comes from an endemic area of the disease with fever and splenomegaly, especially when superimposed on a background of an immunocompromised state.

## 2. Case Report

**Figure 1:**
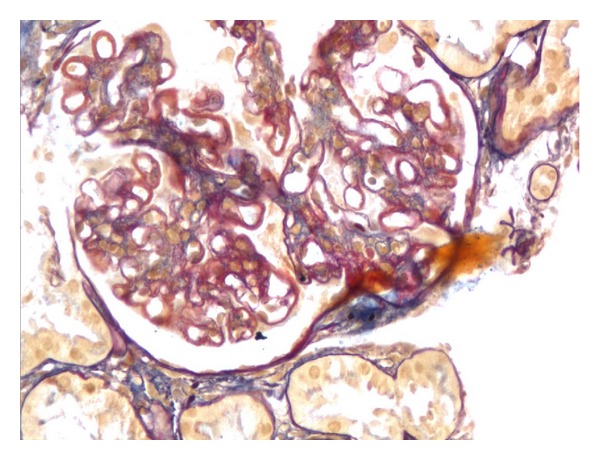
Acid fuchsin or Masson's trichome staining showing reddish coloured deposits on basement membrane.

**Figure 2:**
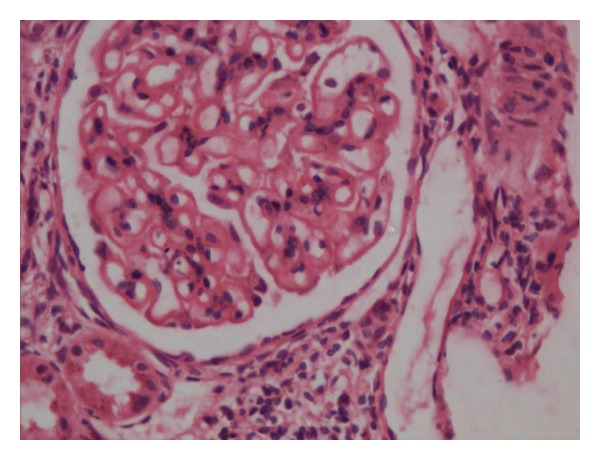
Renal biopsy showing glomerular basement membrane thickening with mesangial proliferation.

**Figure 3:**
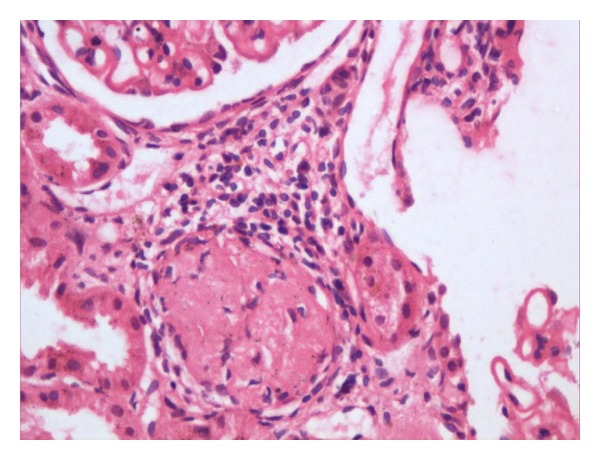
Mononuclear cell infilteration with sclerosed, hyalinised glomeruli indicating chronic disease.

**Figure 4:**
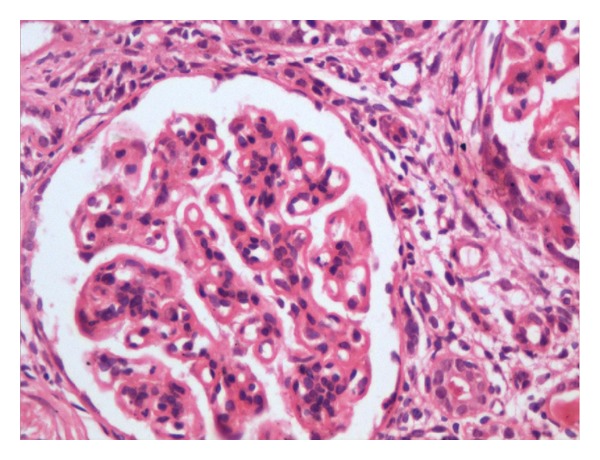
Giant mononuclear cells with pyknosis of nuclei indicating active disease process.

A 30-years-old female presented with complaints of low grade fever, multiple painful small joints with periorbital puffiness and bilateral pedal swelling for one month. She had history of distal phalangeal amputation 1 year back for bilateral upper limb digital infarcts. General examination revealed pallor and bilateral pitting pedal oedema. Palpable spleen 2 cm below subcostal margin was the only significant systemic finding. Haematological investigations revealed haemoglobin 2.56 mmol/L, total leukocyte count 3.4 × 10^9^/L, platelets 59 × 10^9^/L and, albumin : globulin (A : G) ratio 2.0 : 5.6. Renal, liver, and thyroid function tests were normal. 24 hours urinary protein excretion was 581 mg. Antinuclear antibodies (ANA) and anti-double-stranded DNA (Anti dsDNA) antibodies were positive, titres being 7.3 and 6.8 times of upper limit of normal range. Anti-Ro, anti-La, and p-ANCA were also positive. Lupus anticoagulant (LA) and anticardiolipin antibody (ACLA) were negative. Renal biopsy was suggestive of lupus nephritis of mixed type (grade v and ii, predominantly membranous, and mesangial cell proliferation, Figures 1, 2, 3, and 4). She was treated with methylprednisolone pulse therapy followed by oral prednisolone and hydroxychloroquine. One year later, she was again admitted in our ward with history of 1 month of high-grade fever. Low dose of steroids was given without improvement. Liver and spleen was palpable, 3 cm and 10 cm below subcostal margin, respectively, and hepatosplenomegaly was confirmed on ultrasonography. Pancytopenia, A : G reversal and slightly raised autoantibody titres still persisted. Deterioration of patient despite immunosuppressive therapy along with massive splenomegaly, normal C3 and C4, and reduced 24 hour urinary protein, almost ruled out an SLE flare. These features coupled with residence in endemic zone led to a high suspicion of kala azar. Immunochromatographic dipstick test (rK-39) and bone marrow aspiration for LD bodies was negative. Patient did not give the consent for splenic aspiration. However, Direct Agglutination Test (DAT, titre 1 : 1600) and PCR were positive for kala-azar. Patient was started on amphotericin B deoxycholate infusion following which rapid resolution of fever and hepatosplenomegaly was noticed along with improvement in haematological parameters.

## 3. Discussion

Visceral leishmaniasis (VL) has already been reported with SLE [4, 5]. In such cases, it mimics a lupus flare [6]. IL-10 secreted by T-cells promotes B cell survival and plasma cell differentiation. Due to polyclonal activation of B cells, there is formation of several autoantibodies like ANA, and others [2]. Presence of ANA and anti-dsDNA can mislead the diagnosis of SLE in a patient of VL and immunosuppression, as a part of therapy of SLE can be detrimental in these patients. So development of fever, pancytopenia, and splenomegaly due to VL in a patient of SLE on immunosuppressive therapy can mimic disease flare which can lead to increase in dose of immunosuppressive therapy. In our patient fever, small joint pain, facial puffiness, past history of digital infarcts, pancytopenia, positivity of ANA, anti-dsDNA, proteinuria, and biopsy proven lupus nephritis lead to primary diagnosis of SLE. Initially, patient showed slight improvement on steroid therapy but gradual deterioration followed over next few months. Moreover, the derangement in laboratory parameters persisted after 1 year of therapy, along with massive splenomegaly. The suspicion for VL was so high that we went for DAT and PCR despite negative results in rK-39 test and bone marrow studies. This was not the case with earlier reports in which VL was diagnosed easily with the latter tests. Therefore, we conclude that a high suspicion of kala-azar should always be kept in a patient of SLE, especially when he comes from an endemic area, and immunosuppressive therapy should be started only after ruling it out diligently.
